# Predicting radiation pneumonitis with dose-segmented radiomics in locally advanced non- small cell lung cancer patients undergoing consolidative immunotherapy post- concurrent chemoradiotherapy

**DOI:** 10.3389/fimmu.2025.1684629

**Published:** 2025-09-26

**Authors:** Weiqing Wang, Yanan Wang, Xiaohan Wang, Jinming Yu, Xue Meng

**Affiliations:** ^1^ The First Affiliated Hospital of Shantou University Medical College, Shantou, Guangdong, China; ^2^ Department of Radiation Oncology, Shandong Cancer Hospital and Institute, Shandong First Medical University and Shandong Academy of Medical Sciences, Jinan, Shandong, China; ^3^ School of Public Health, Shandong First Medical University and Shandong Academy of Medical Sciences, Jinan, China

**Keywords:** radiation pneumonitis, LA-NSCLC, consolidative immunotherapy, CT radiomics, machine learning

## Abstract

**Objective:**

To develop and validate a machine learning model that integrates dose distribution-based radiomics, clinicopathological parameters, and hematological inflammatory biomarkers for predicting radiation pneumonitis (RP) in locally advanced non-small cell lung cancer (LA-NSCLC) patients receiving immuno-consolidation therapy after concurrent chemoradiation (CCRT).

**Methods:**

This retrospective study analyzed 161 locally advanced non-small cell lung cancer (LA-NSCLC) patients divided into training (n=112) and validation (n=49) cohorts. Radiomics features were extracted from planning CT scans across nine 5-Gy dose gradients (0–60 Gy), including the initial positioning CT (before radiotherapy) and a resetting CT (after a cumulative dose of 40–50 Gy), all within regions of interest (ROIs). Longitudinal feature changes were analyzed, followed by LASSO-based feature selection and logistic regression modeling. Machine learning methods evaluated associations between radiomics signatures (RS), clinical features, hematological inflammatory markers, and RP. Model performance was evaluated with AUC metrics and decision curve analysis (DCA).

**Results:**

Radiomics signatures across dose ranges (RS1:5 Gy; RS3:10–15 Gy; RS4:15–20 Gy; RS5:20–30 Gy; RS7:40–50 Gy; RS8:50–55 Gy; RS9:55–60 Gy) were developed. RS8 demonstrated the highest validation AUC (0.854). The model based on RS8 combined with tumor location achieved an AUC of 0.918 in the training cohort for predicting RP, whereas the addition of the neutrophil-to-lymphocyte ratio at 4 week (NLR 4w) to this model resulted in a marginally higher AUC of 0.938.

**Conclusions:**

The combined model improves RP prediction in LA-NSCLC patients undergoing post-CCRT consolidative immunotherapy, offering a novel approach for personalized patient management.

## 1 Introduction

Radiotherapy (RT) serves as the primary therapeutic approach for individuals with unresectable locally advanced non-small cell lung cancer (LA-NSCLC) (1). Following the outcomes of the PACIFIC trial, anti-PD-L1 therapies subsequent to radical chemo-radiotherapy have been established as the standard care for this condition, demonstrating a notable survival advantage (2, 3). Despite the promising synergy between immune checkpoint inhibitors (ICI) and RT, awareness of the risk of radiation pneumonitis (RP), a frequent adverse effect, is crucial. The incidence of any-grade pneumonitis or RP was 33.9% and 24.8%, respectively, among patients treated with durvalumab versus those on a placebo regimen in the PACIFIC trial (2).This treatment regimen has also been associated with an increased incidence of pneumonia and more frequent treatment interruptions in a subset of patients (2), potentially reducing clinical benefits (4). Hence, accurately predicting RP is vital for optimizing RT dosing to maximize therapeutic effects while minimizing RP risk.

Currently, RP predictive models are predominantly based on clinical factors and dosimetric parameters, including age, gender, smoking history, tumor location, pre-existing pulmonary comorbidities, and concurrent chemotherapy treatment (5–8). Dosimetric predictors, such as the volume of lung receiving 5 Gy (V5), volume of lung receiving 20 Gy (V20), mean lung dose (MLD), total lung radiation dose, and daily radiation dose, are well-recognized (6, 9–11). The normal tissue complication probability (NTCP) model, which utilizes comprehensive data from the dose-volume histogram (DVH), has shown superior predictive performance over dosimetric factors alone (12).

Radiomics, an emerging field, converts conventional medical images into quantifiable data for mining and subsequent analysis, supporting clinical decision-making. Its role in predicting clinically significant RP has attracted considerable attention. Predictive models based on radiomic features have become increasingly used to identify patients at high risk for RP (13, 14). Studies have indicated that incorporating dosiomic (dose-based radiomic) features can improve the predictive efficacy beyond DVH or NTCP models alone for RP prediction (15, 16). Additionally, the integration of radiomics and dosiomic features into dual-omics models has been shown to further enhance the predictive capability for RP (14).

Recent developments in multi-omics, combining radiomics with hematological inflammatory markers, dosimetry, and clinical features, have demonstrated exceptional predictive performance (17). Nevertheless, there exists a scarcity of radiomic data on risk factors for predicting RP occurrence in LA-NSCLC patients who received consolidative immunotherapy after CCRT compared to current RP models. Addressing this gap by developing new prognostic models with improved predictive ability is crucial for optimizing the therapeutic benefits of current RT and ICI regimens while minimizing RP incidence. In this study, we introduce a composite predictive model that integrates radiomics based on CT dose distribution, clinical parameters, and hematological inflammatory biomarkers to predict RP in LA-NSCLC patients.

## 2 Materials and methods

### 2.1 Study population and data characteristics

**Figure 1:**
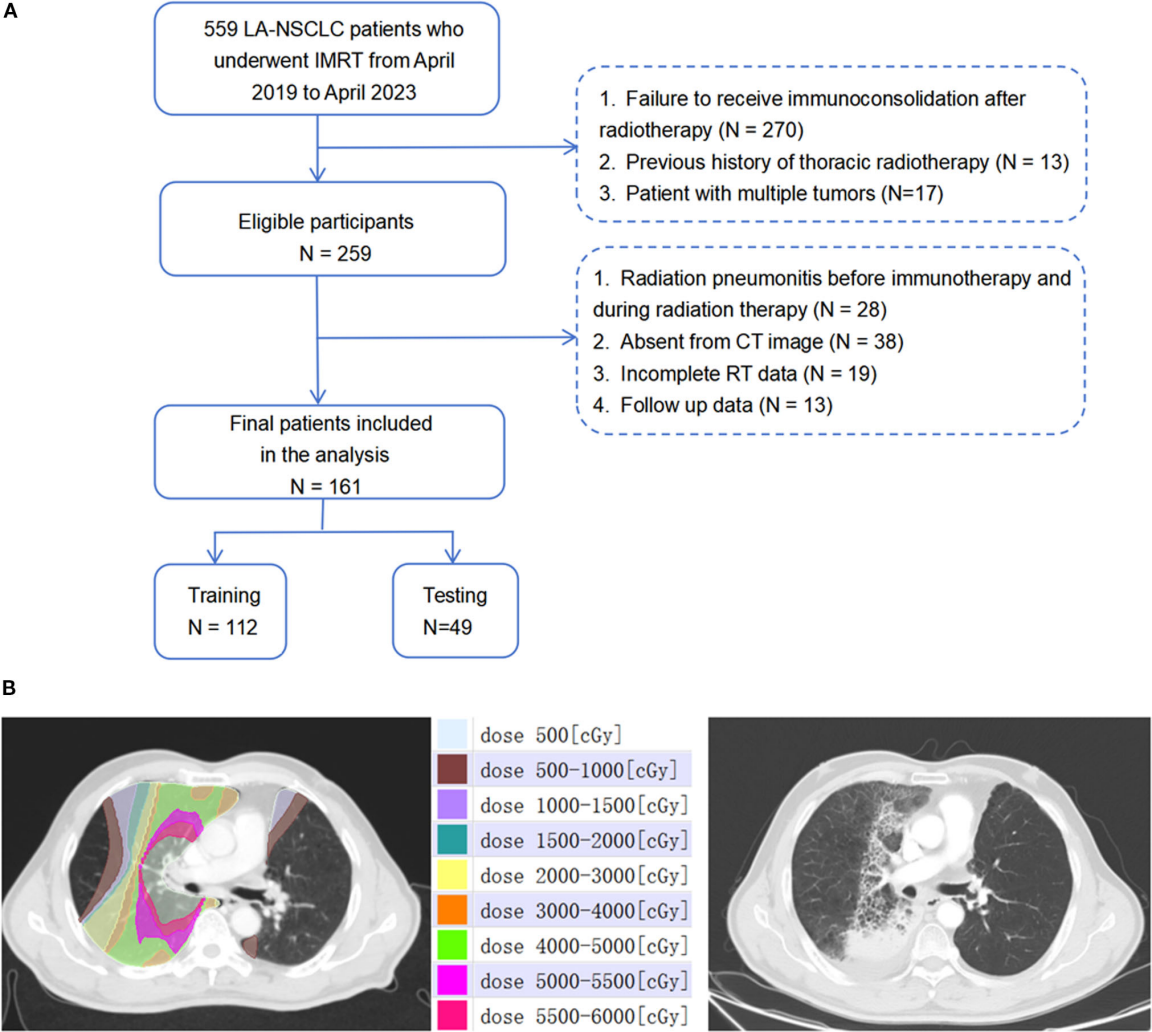
**(A)** The workflow of schematic diagram of data collection of RP≥2. **(B)** Dose-distribution-based CT images before radiotherapy and representative images of patients presenting with grade 3 RP after radiotherapy.1Gy=100cGy.

This retrospective study enrolled a total of 161 patients diagnosed with Locally Advanced Non-Small Cell Lung Cancer (LA-NSCLC) at the Shandong Cancer Hospital. These patients received consolidative immunotherapy following RT from April 2019 to April 2023 (see **Figure 1A**). The inclusion criteria were: (1) pathologically confirmed stage IIIA-IIIC NSCLC according to the AJCC 8th edition; (2) completion of curative-intent thoracic RT ranging from 50–70 Gy; (3) undergoing concurrent chemoradiotherapy followed by at least two cycles of consolidative immunotherapy; (4) observation of RP within six months post-RT. Patients were excluded based on the following: (1) occurrence of RP prior to consolidative immunotherapy; (2) absence of diagnostic imaging at the time of RP diagnosis; (3) a previous history of thoracic RT; (4) incomplete RT data, including those not undergoing reset CT during RT; (5) diagnosis of immune-related pneumonitis. Baseline clinicopathologic and dosimetric data are summarized in **Table 1**.

**Table 1:** Baseline characteristics of the patients.

Characteristic		Training cohort (N=112)			Validation cohort (N=49)		
		Non-RP, N=78	RP, N=34	p-value	Non-RP, N=33	RP, N=16	p-value
Gender				0.488			>0.999
	Male	72 (92%)	30 (88%)		30 (91%)	15 (94%)	
	Female	6 (7.7%)	4 (12%)		3 (9.1%)	1 (6.3%)	
Age(years)				0.285			0.726
	60	24 (31%)	14 (41%)		9 (27%)	3 (19%)	
	60	54 (69%)	20 (59%)		24 (73%)	13 (81%)	
Histology				0.435			0.520
	AC	24 (31%)	8 (24%)		9 (27%)	6 (38%)	
	SCC	54 (69%)	26 (76%)		24 (73%)	10 (63%)	
Smoking History				0.525			>0.999
	No	25 (32%)	13 (38%)		6 (18%)	3 (19%)	
	Yes	53 (68%)	21 (62%)		27 (82%)	13 (81%)	
Tumor Location				<0.001			0.079
	Upper	60 (77%)	5 (15%)		17 (52%)	4 (25%)	
	Middle or Lower	18 (23%)	29 (85%)		16 (48%)	12 (75%)	
Pulmonary comorbidities				0.001			0.261
	No	61 (78%)	16 (47%)		22 (67%)	8 (50%)	
	Yes	17 (22%)	18 (53%)		11 (33%)	8 (50%)	
TNM stage				0.072			0.653
	IIIA	25 (32%)	11 (32%)		14 (42%)	5 (31%)	
	IIIB	35 (45%)	21 (62%)		13 (39%)	9 (56%)	
	IIIC	18 (23%)	2 (5.9%)		6 (18%)	2 (13%)	
KPS score				0.486			0.266
	80	33 (42%)	12 (35%)		13 (39%)	9 (56%)	
	90	45 (58%)	22 (65%)		20 (61%)	7 (44%)	
Immunotherapeutic drugs				0.509			0.363
	PD-1	36 (46%)	18 (53%)		14 (42%)	9 (56%)	
	PD-L1	42 (54%)	16 (47%)		19 (58%)	7 (44%)	
PD-L1 expression				0.317			0.378
	>50%	9 (12%)	1 (2.9%)		5 (15%)	1 (6.3%)	
	1–49%	9 (12%)	7 (21%)		4 (12%)	2 (13%)	
	<1%	11 (14%)	6 (18%)		2 (6.1%)	4 (25%)	
	Unavailable	49 (63%)	20 (59%)		22 (67%)	9 (56%)	
BMI	Mean (SD)	23.90 (3.54)	24.66 (2.91)	0.243	24.11 (2.86)	24.95 (3.62)	0.301
	Median (Q1, Q3)	23.88 (21.30, 26.12)	24.79 (22.83, 26.57)		23.53 (22.23, 26.13)	24.36 (23.29, 25.66)	
RS8	Mean (SD)	-1.32 (0.63)	-0.09 (1.01)	<0.001	-1.50 (0.65)	-0.51 (0.70)	<0.001
	Median (Q1, Q3)	-1.29 (-1.78, -0.87)	-0.02 (-0.92, 0.44)		-1.53 (-1.89, -1.17)	-0.39 (-0.91, -0.24)	
MLD		1,044.90 (796.30, 1,244.70)	1,014.90 (865.20, 1,282.10)	0.413	1,127.40 (865.10, 1,289.20)	954.70 (729.45, 1,189.05)	0.090
V5		35.64 (30.20, 47.42)	38.57 (30.50, 47.58)	0.461	37.53 (31.28, 47.38)	34.82 (24.61, 39.07)	0.156
V20		18.77 (14.18, 23.48)	17.98 (15.24, 22.70)	0.692	20.45 (14.81, 22.60)	17.03 (12.36, 22.27)	0.164
V30		13.10 (9.33, 16.29)	12.28 (10.46, 16.31)	0.773	15.20 (10.68, 17.26)	11.95 (7.48, 15.42)	0.099
V40		8.46 (5.40, 10.54)	8.53 (5.25, 11.56)	0.529	9.45 (6.92, 12.09)	7.45 (4.06, 11.06)	0.171
RT Dose(cGy)		6,000.00 (5,940.00, 6,000.00)	6,000.00 (5,600.00, 6,000.00)	0.597	6,000.00 (6,000.00, 6,000.00)	6,000.00 (5,900.00, 6,000.00)	0.780
PTV/LV		0.08 (0.06, 0.12)	0.09 (0.05, 0.12)	0.730	0.08 (0.05, 0.11)	0.06 (0.03, 0.09)	0.479

### 2.2 Treatment protocol

**Figure 2:**
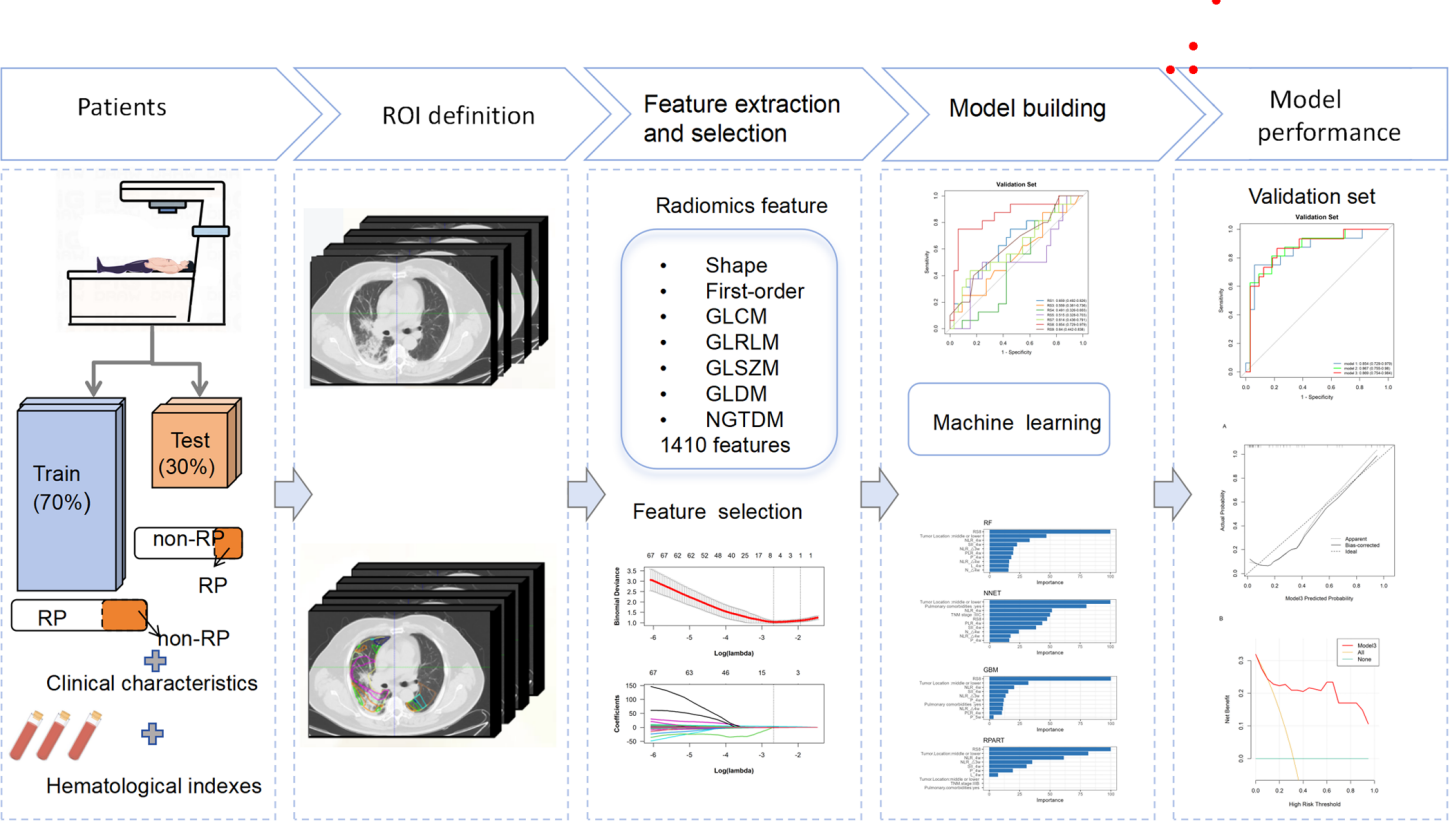
An overall workflow of risk analysis of RP≥2.

Patients were treated using intensity-modulated radiotherapy (IMRT), with RT plans generated using the Eclipse system (Varian Medical Systems, Palo Alto, CA, version 13.5.35). Prescribed RT doses varied from 50 Gy to 66 Gy, delivered in fractions of 1.8 Gy to 2 Gy, once daily, five days a week. Patients who did not exhibit disease progression or persistent RT toxicity commenced up to 12 months of consolidative immunotherapy within 60 days following the completion of concurrent chemoradiotherapy (CCRT). Chemotherapy regimens were tailored by the attending medical oncologists. The immunotherapy regimens included sintilimab and durvalumab. The study population was randomly divided into a training set (n=112) and a validation set (n=49) at an approximate 7:3 ratio. The workflow of the study is depicted in **Figure 2**.

### 2.3 RP evaluation

Follow-up for all cases was conducted for at least six months post-RT, focusing on the incidence of RP. A senior radiologist and oncologist evaluated RP occurrences. RP was identified based on: (1) a history of RT; (2) symptoms such as shortness of breath, low-grade fever, and a dry cough; (3) thoracic CT imaging findings within the high-dose radiation field that diverged from normal lobar anatomy, showing patches of solid lesions (18). Patients experiencing RP events, categorized as grade ≥2, were evaluated using the Common Terminology Criteria for Adverse Events version 5.0 (CTCAE V5.0). RP monitoring encompassed clinical examinations, symptom assessment, medical record review, laboratory tests, and regular follow-up CT scans at 1, 3, and 6 months after RT completion, followed by at least every three months during the first year.

### 2.4 Collection and definition of parameters

Clinical information, imaging data, and results from laboratory tests were compiled from our institution’s medical records. Clinicopathological parameters such as age, gender, smoking history, comorbidities, pathological diagnosis, tumor stage and location, radiation dose, and PD-L1 expression levels were gathered from data collected before the initiation of RT. Inflammatory markers, including the Platelet-to-Lymphocyte Ratio (PLR), Neutrophil-to-Lymphocyte Ratio (NLR), Lymphocyte-to-Monocyte Ratio (LMR), and Systemic Immune-Inflammation Index (SII), were assessed at six different intervals: one week prior to RT commencement (as baseline), and at one (1w), two (2w), three (3w), four (4w), and five (5w) weeks during RT. These markers were calculated using the formulas: PLR = platelet count [P]/lymphocyte count [L], NLR = neutrophil count [N]/L, LMR = L/monocyte count [M], and SII = P × N/L. Additionally, dosimetric parameters were extracted from the Varian Treatment Planning System, contributing to our analysis of treatment characteristics. MLD = mean dose of total lungs. PTV/LV= planning target volume/total lungs volume. Vxx = % of the whole OAR receiving ≥xx Gy. V5 = percentage of total lungs volume receiving 5 Gy. V20 = percentage of total lungs volume receiving 20 Gy. V30 = percentage of total lungs volume receiving 30 Gy. V40 = percentage of total lungs volume receiving 40 Gy.

### 2.5 CT image acquisition and ROI identification

Contrast-enhanced treatment planning CT images were obtained utilizing a Philips Brilliance Big Bore CT scanner (Philips Medical Systems, Inc, Cleveland, OH), following a standardized clinical protocol. The scanning parameters were as follows: tube voltage, 120 kVp; tube current, 200 mA; slice thickness, 3mm. These images included the initial positioning CT scan before radiation therapy (CT_1_) and a subsequent scan after a cumulative dose of 40 to 50 Gy (CT_2_), both exported in the Digital Imaging and Communications in Medicine (DICOM) format. Both CT_1_ and CT_2_ are simulation CT scans acquired using the same CT scanner, with the patient maintained in the same position during the operation. The Eclipse treatment planning system (version 13.5.35; Varian Medical Systems, Palo Alto, CA) was utilized to categorize dose ranges into nine distinct zones: 0–5 Gy, 5–10 Gy, 10–15 Gy, 15–20 Gy, 20–30 Gy, 30–40 Gy, 40–50 Gy, 50–55 Gy, and 55–60 Gy. Corresponding regions of interest (ROIs) for each dose zone were then identified, with a focus on maintaining a minimum volume of ≥0.5 cm^3^ for individual ROIs. **Figure 1B** showcases representative CT images illustrating patient-specific dose distribution.

These images, along with dose distribution diagrams and RT plans, were imported into the MedMind Technology Imaging System. A rigid registration was performed to align the resetting CT images with the initial planning positioning CT images. Precise alignment was ensured between the positioning CT images and the subsequent resetting CT to maintain uniformity in spatial coordinates and shape across both scans. Two radiation oncologists, with 11 and 16 years of experience in radiotherapy-related image analysis respectively, manually corrected any registration errors exceeding 1mm. Displacement vector fields were applied to adapt the three-dimensional dose distribution associated with the planned CT, ensuring the spatial consistency of ROIs across different dose gradients before and after RT.

### 2.6 Extraction, selection, and analysis of texture features

Texture features were extracted using the MedMind Technology Imaging System, with initial image discretization set at a fixed bin width of 25 to standardize the analysis. A comprehensive set of 1810 features spanning seven distinct radiomics feature classes were extracted, as detailed in **Supplementary Material Table S1**.

Prior to feature extraction, a normalization procedure (z-score transformation) standardized the CT values across all images, ensuring a uniform intensity range. The ROIs were semi-automatically delineated on the CT images of 40 randomly selected patients by two expert radiologists. The intragroup correlation coefficient (ICC) was calculated to assess the reliability of the extracted features, with those achieving an ICC greater than 0.8 being selected for further analysis. Feature change (ΔRF) was quantified as ΔRF = [(RF_CT2_ - RF_CT1_)/RF_CT1_] × 100%, where RF_CT1_ and RF_CT2_ represent the radiomic features extracted from the initial and subsequent CT scans, respectively. Features with missing values were excluded, and the analysis proceeded with the remaining data.

The least absolute shrinkage and selection operator (LASSO) algorithm was applied to refine the feature selection across the nine ROIs, optimizing the selection process through a 10-fold cross-validation method to determine the most suitable lambda (λ) value. The methodological approach for feature selection using the LASSO algorithm is illustrated in **Supplementary Figure S1** in the **Supplementary Material**. A radiomics signature (RS) was constructed utilizing a linear combination of the selected features weighted by their LASSO coefficients.

### 2.7 Construction and validation of radiomics signatures

To construct the RS models, we employed logistic regression (LR) classifier to identify the most effective ones for constructing ROI-specific radiomics features across different dose regions. The validation phase employed a rigorous procedure involving grid search and cross-validation to fine-tune the hyperparameters and enhance model performance.

Receiver operating characteristic (ROC) analysis was employed to evaluate the performance of each model, utilizing the area under the ROC curve (AUC) as the principal measure of efficacy. LR iteratively determines the most potent linear mix of variables that optimally predicts the observed outcome, leveraging the linear regression components on the logit scale. LR was implemented by the R package “glmnet”. In the training process, the optimal hyperparameters were identified through the application of grid search and ten-fold cross-validation procedures. This comprehensive approach aimed to establish a robust framework for the development and validation of predictive radiomics signatures.

### 2.8 Construction and validation of combined model

Variables with p-value less than 0.1 in baseline characteristics (**Tables 1**, **2**) were selected. The importance of each variable in the RP prediction was selected using a ten-fold cross-validation method by ranking the importance of each variable among the four models, namely, Random Forest (RF), Neural Network (NNET), Gradient Boosting Machine (GBM), and Recursive Partitioning and Regression Trees (RPART) to rank the importance of each variable and filter out the stable-performing variables in RP prediction. Subsequently, logistic regression models were employed to evaluate the predictive efficacy of the combined top three stable variables for RP. The calibration curve was also generated to evaluate the concordance between the predicted and observed RP probabilities. Furthermore, decision curve analysis (DCA) was performed to ascertain the clinical applicability of the combined model.

**Table 2:** Hematological parameters of the patients.

Characteristic		Training cohort (N=112)			Validation cohort (N=49)		
		Non-RP, N=78	RP, N=34	p-value	Non-RP, N=33	RP, N=16	p-value
N	baseline	3.98 (2.48, 5.19)	3.28 (2.18, 4.68)	0.263	3.40 (2.62, 4.63)	3.70 (3.12, 4.50)	0.536
	1w	3.55 (2.13, 5.06)	3.41 (2.09, 5.12)	0.630	2.84 (2.06, 3.81)	3.56 (2.29, 7.45)	0.089
	2w	2.79 (1.86, 4.02)	3.23 (1.99, 4.23)	0.310	2.33 (1.73, 3.49)	2.44 (2.07, 3.49)	0.577
	3w	2.97 (2.14, 4.49)	3.08 (2.18, 4.60)	0.734	2.66 (1.77, 4.58)	3.61 (2.15, 4.17)	0.533
	4w	2.88 (2.15, 3.43)	3.04 (2.24, 4.16)	0.467	2.87 (2.23, 3.22)	3.04 (2.63, 4.01)	0.144
	5w	2.79 (2.24, 4.12)	3.44 (2.41, 4.66)	0.334	3.38 (2.39, 4.35)	2.86 (2.19, 4.05)	0.654
	△2w	-1.52 (-2.61, 0.41)	-0.32 (-1.27, 0.67)	0.093	-0.70 (-2.17, 0.11)	-0.86 (-2.20, -0.07)	0.922
	△3w	-1.00 (-2.99, 0.72)	-0.39 (-1.38, 1.13)	0.107	-0.77 (-2.08, 0.13)	-0.94 (-1.26, 0.46)	0.571
	△4w	-0.89 (-2.53, 1.12)	0.07 (-1.48, 1.66)	0.081	-0.92 (-2.17, 0.90)	-0.98 (-1.50, 0.44)	0.801
	△5w	-0.86 (-2.49, 1.11)	0.11 (-2.08, 2.06)	0.212	-0.46 (-1.88, 1.82)	-1.09 (-1.76, 1.01)	0.759
L	baseline	1.64 (1.27, 1.98)	1.59 (1.37, 2.24)	0.924	1.66 (1.36, 1.90)	1.53 (1.38, 1.81)	0.488
	1w	1.14 (0.84, 1.56)	1.23 (1.02, 1.50)	0.519	1.23 (0.88, 1.60)	0.87 (0.75, 1.30)	0.093
	2w	0.90 (0.71, 1.15)	1.01 (0.72, 1.28)	0.425	0.89 (0.79, 1.25)	0.80 (0.59, 1.12)	0.230
	3w	0.74 (0.55, 1.02)	0.76 (0.49, 0.94)	0.625	0.86 (0.68, 1.09)	0.62 (0.45, 0.78)	0.015
	4w	0.70 (0.53, 0.92)	0.56 (0.46, 0.80)	0.068	0.79 (0.71, 0.97)	0.59 (0.50, 0.62)	<0.001
	5w	0.59 (0.42, 0.87)	0.61 (0.45, 0.76)	0.923	0.73 (0.49, 0.94)	0.50 (0.28, 0.68)	0.026
	△2w	-0.64 (-1.06, -0.35)	-0.67 (-0.92, -0.14)	0.716	-0.54 (-1.07, -0.33)	-0.74 (-0.91, -0.36)	0.902
	△3w	-0.82 (-1.27, -0.52)	-0.94 (-1.09, -0.50)	0.806	-0.84 (-1.09, -0.46)	-0.89 (-1.15, -0.55)	0.430
	△4w	-0.90 (-1.18, -0.60)	-1.01 (-1.43, -0.56)	0.456	-0.74 (-1.10, -0.46)	-0.98 (-1.26, -0.85)	0.113
	△5w	-1.02 (-1.29, -0.69)	-0.93 (-1.43, -0.54)	0.674	-0.91 (-1.21, -0.55)	-1.02 (-1.18, -0.72)	0.540
R	baseline	4.27 (3.81, 4.63)	4.27 (4.03, 4.66)	0.669	4.25 (3.89, 4.60)	4.23 (3.72, 4.55)	0.677
	1w	4.20 (3.79, 4.40)	4.25 (3.87, 4.57)	0.506	4.24 (3.69, 4.47)	3.99 (3.53, 4.26)	0.362
	2w	4.02 (3.63, 4.33)	4.18 (3.68, 4.49)	0.418	4.03 (3.55, 4.29)	3.79 (3.52, 4.09)	0.304
	3w	4.03 (3.68, 4.30)	4.10 (3.74, 4.56)	0.505	4.00 (3.60, 4.25)	3.60 (3.35, 4.04)	0.247
	4w	3.95 (3.49, 4.36)	3.95 (3.65, 4.49)	0.469	4.02 (3.50, 4.29)	3.59 (3.20, 3.96)	0.259
	5w	3.96 (3.48, 4.29)	3.99 (3.55, 4.34)	0.413	4.07 (3.44, 4.45)	3.62 (3.47, 4.11)	0.149
	△2w	-0.23 (-0.43, 0.07)	-0.14 (-0.26, -0.01)	0.408	-0.31 (-0.50, -0.12)	-0.26 (-0.63, -0.08)	0.844
	△3w	-0.27 (-0.46, -0.08)	-0.18 (-0.48, 0.01)	0.576	-0.37 (-0.62, -0.05)	-0.50 (-0.73, -0.20)	0.238
	△4w	-0.27 (-0.51, -0.02)	-0.28 (-0.53, 0.01)	0.927	-0.38 (-0.63, -0.11)	-0.57 (-0.69, -0.17)	0.304
	△5w	-0.37 (-0.78, -0.15)	-0.25 (-0.62, 0.00)	0.327	-0.32 (-0.62, -0.02)	-0.36 (-0.82, -0.10)	0.431
P	baseline	238.00 (184.00, 310.00)	250.00 (192.00, 333.00)	0.606	201.00 (169.00, 248.00)	250.50 (200.00, 292.00)	0.115
	1w	192.50 (155.00, 283.00)	200.00 (153.00, 276.00)	0.751	189.00 (156.00, 234.00)	226.00 (153.00, 267.00)	0.374
	2w	185.00 (141.50, 232.50)	198.50 (166.50, 257.50)	0.226	165.00 (137.00, 209.50)	176.00 (142.00, 229.00)	0.314
	3w	174.00 (133.00, 213.00)	185.00 (139.00, 241.00)	0.181	175.00 (138.00, 225.00)	167.00 (117.00, 245.00)	0.991
	4w	166.00 (135.00, 213.00)	182.50 (168.00, 235.50)	0.068	173.50 (121.50, 219.00)	151.00 (123.00, 221.00)	0.632
	5w	169.50 (128.00, 227.00)	186.50 (147.00, 228.00)	0.077	165.00 (135.00, 195.50)	169.00 (150.00, 212.50)	0.274
	△2w	-46.50 (-130.50, -0.50)	-35.50 (-126.00, 6.00)	0.685	-40.00 (-100.50, 4.50)	-46.50 (-93.50, -21.50)	0.710
	△3w	-59.00 (-129.00, -22.00)	-49.00 (-100.00, -11.00)	0.402	-63.00 (-89.00, -5.00)	-57.00 (-115.00, -16.00)	0.470
	△4w	-62.00 (-126.00, -22.00)	-71.50 (-119.00, -27.00)	0.736	-55.50 (-105.50, -4.00)	-54.00 (-158.00, -26.00)	0.299
	△5w	-77.50 (-148.00, -12.50)	-65.50 (-122.00, -7.00)	0.473	-65.00 (-88.00, 4.50)	-51.00 (-70.00, -29.50)	0.956
PLR	baseline	142.72 (115.11, 203.97)	177.36 (122.50, 218.64)	0.240	131.35 (94.47, 159.43)	157.30 (136.18, 215.82)	0.053
	1w	177.84 (127.61, 241.98)	177.24 (120.38, 236.99)	0.811	152.27 (126.72, 183.33)	196.15 (133.77, 370.00)	0.087
	2w	199.52 (129.06, 285.39)	214.06 (126.51, 266.67)	0.807	171.59 (134.67, 227.96)	217.74 (173.23, 318.74)	0.054
	3w	226.92 (158.02, 290.70)	266.47 (192.86, 365.31)	0.103	197.44 (159.56, 280.91)	253.03 (190.59, 342.31)	0.068
	4w	225.26 (173.96, 318.60)	312.80 (219.50, 413.42)	0.015	193.90 (140.10, 273.82)	253.23 (214.52, 424.14)	0.004
	5w	292.97 (200.00, 434.49)	327.84 (255.00, 402.22)	0.296	219.06 (181.50, 313.06)	374.49 (316.37, 562.06)	<0.001
	△2w	82.17 (6.96, 239.62)	65.22 (-3.33, 189.39)	0.919	42.20 (-18.18, 118.52)	66.35 (43.45, 160.04)	0.199
	△3w	84.13 (21.43, 227.13)	51.43 (3.47, 128.38)	0.117	33.17 (1.65, 153.57)	61.29 (14.16, 138.18)	0.567
	△4w	57.94 (13.89, 157.28)	81.63 (31.35, 123.59)	0.943	46.26 (-6.37, 161.65)	59.34 (20.63, 160.55)	0.314
	△5w	73.77 (15.62, 143.09)	87.18 (12.28, 152.78)	0.930	68.88 (-5.00, 158.63)	53.97 (33.48, 70.91)	0.689
NLR	baseline	2.43 (1.60, 3.56)	2.20 (1.41, 3.08)	0.435	2.24 (1.48, 2.80)	2.42 (1.70, 3.81)	0.257
	1w	2.89 (1.87, 4.68)	2.80 (1.48, 4.65)	0.409	2.64 (1.42, 3.56)	3.62 (2.67, 11.03)	0.036
	2w	2.89 (1.95, 4.65)	3.04 (2.05, 4.99)	0.761	2.58 (1.71, 4.13)	3.23 (2.42, 4.04)	0.224
	3w	3.78 (2.61, 7.14)	4.04 (2.56, 7.26)	0.734	3.55 (2.01, 4.95)	4.98 (3.47, 6.84)	0.050
	4w	3.63 (3.18, 4.97)	4.14 (3.70, 6.08)	0.021	3.39 (2.83, 3.66)	5.87 (4.17, 7.48)	<0.001
	5w	5.24 (3.30, 9.23)	4.80 (3.71, 6.64)	0.667	5.46 (3.07, 7.81)	5.83 (3.50, 14.98)	0.219
	△2w	1.66 (-0.72, 4.91)	1.05 (-0.30, 3.44)	0.607	1.65 (-0.25, 3.36)	0.99 (-0.15, 3.30)	0.714
	△3w	1.38 (-1.25, 4.13)	-0.10 (-1.53, 1.05)	0.015	0.95 (-0.43, 2.79)	0.79 (-0.63, 1.53)	0.495
	△4w	0.95 (-1.15, 2.60)	-0.14 (-2.46, 1.88)	0.087	0.94 (-1.10, 2.64)	0.80 (-0.69, 1.69)	0.799
	△5w	0.71 (-0.96, 2.61)	-0.25 (-3.90, 2.48)	0.247	0.51 (-1.44, 2.59)	1.39 (-0.74, 1.78)	0.689
LMR	baseline	3.36 (2.29, 4.47)	3.58 (2.58, 6.27)	0.159	2.88 (2.54, 3.69)	2.88 (2.34, 3.80)	0.681
	1w	3.19 (1.96, 5.60)	3.32 (2.31, 5.04)	0.992	5.67 (3.10, 12.40)	2.41 (1.66, 4.81)	0.014
	2w	2.17 (1.38, 3.79)	2.38 (1.70, 3.12)	0.615	2.08 (1.32, 4.05)	1.92 (1.40, 2.59)	0.355
	3w	1.46 (1.00, 2.84)	1.79 (1.21, 3.45)	0.237	1.66 (1.32, 2.07)	1.21 (1.00, 1.74)	0.168
	4w	1.71 (1.05, 2.44)	1.82 (1.08, 2.53)	0.908	1.98 (1.40, 3.43)	1.24 (0.96, 1.72)	0.004
	5w	1.26 (0.86, 1.80)	1.40 (1.08, 2.00)	0.188	1.27 (0.91, 1.97)	1.15 (0.82, 1.32)	0.246
	△2w	0.43 (-3.12, 3.05)	0.44 (-1.71, 2.13)	0.913	1.00 (-1.85, 3.10)	2.00 (-1.84, 4.24)	0.633
	△3w	0.92 (-1.79, 4.92)	1.64 (-4.22, 5.50)	0.919	1.56 (-1.79, 5.67)	1.83 (-4.19, 9.60)	0.758
	△4w	2.06 (-0.88, 7.38)	0.23 (-7.02, 5.58)	0.200	1.45 (-1.08, 3.56)	3.13 (-4.75, 5.93)	0.409
	△5w	0.80 (-4.08, 5.66)	-1.73 (-6.00, 3.41)	0.269	0.38 (-2.41, 6.33)	2.26 (-15.21, 6.96)	0.673
SII	baseline	557.81 (331.66, 929.11)	583.89 (301.05, 936.00)	0.907	412.03 (304.96, 646.31)	603.30 (451.85, 981.03)	0.090
	1w	576.56 (380.27, 1,039.96)	649.08 (277.28, 1,180.00)	0.738	379.16 (263.54, 743.33)	798.83 (516.47, 1,981.82)	0.019
	2w	538.72 (328.42, 876.71)	569.76 (379.55, 1,069.33)	0.382	413.50 (253.19, 674.64)	563.03 (415.49, 848.05)	0.064
	3w	682.16 (385.42, 1,051.59)	803.36 (477.89, 1,368.34)	0.294	524.27 (395.05, 896.48)	951.62 (535.99, 1,201.02)	0.075
	4w	602.74 (412.19, 1,131.24)	807.04 (706.78, 1,241.00)	0.021	553.03 (429.32, 775.56)	921.74 (589.92, 1,071.49)	0.002
	5w	830.96 (532.35, 1,639.99)	1,041.67 (649.91, 1,863.21)	0.463	861.91 (499.51, 1,243.40)	947.16 (774.50, 2,426.09)	0.099
	△2w	-40.74 (-400.91, 35.01)	-72.53 (-226.83, -10.37)	0.576	1.44 (-84.32, 79.20)	-23.26 (-165.82, 7.29)	0.379
	△3w	-67.50 (-270.34, 89.59)	-12.15 (-92.19, 41.74)	0.340	-13.03 (-88.64, 37.77)	-21.24 (-156.07, 12.83)	0.552
	△4w	-23.17 (-192.83, 83.46)	-15.33 (-180.86, 70.91)	0.807	-24.18 (-198.33, 93.29)	-20.84 (-183.53, 11.33)	0.694
	△5w	-52.45 (-314.15, 33.68)	-5.10 (-126.00, 60.32)	0.184	2.72 (-99.70, 83.05)	-38.79 (-127.71, 6.00)	0.416

### 2.9 Statistical analysis

The study’s statistical analysis encompassed clinical, dosimetric, and hematological inflammatory parameters. Continuous variables were analyzed utilizing either the Mann-Whitney U test or the independent-samples T test, depending on their distribution, while chi-square tests were utilized for categorical variables to determine significance levels.

Clinical characteristics with substantial missing data were excluded to ensure the integrity of the analysis. The LASSO algorithm was conducted using the “glmnet” package in R, facilitating the selection of relevant features. Calculations of the area under the receiver operating characteristic curve (AUC) along with its 95% confidence interval were conducted employing the ‘pROC’ package, aiding in the evaluation of model performance. All statistical analyses were carried out using R software (version 4.3.0).

## 3 Results

### 3.1 Characteristics of study participants

**Tables 1** and **2** detail the clinical, dosimetric, and hematological characteristics of the study participants. Analyses revealed no significant differences between the RP and non-RP groups in terms of age, gender, pathological type, smoking history, TNM stage, Karnofsky Performance Status (KPS) score immunotherapy regimens, PD-L1 expression and BMI, all yielding p-values greater than 0.05. However, within the training set, statistically significant differences were observed in tumor location, pulmonary comorbidities, and the radiomics signature RS8 (*p*<0.05). Additionally, among the hematological inflammation parameters, PLR at 4 weeks (PLR4w), NLR at 4 weeks (NLR 4w), NLR △3w (the ratio of change at 3 weeks) and SII at 4 weeks (SII 4w) showed significant differences in the training set (*p*<0.05).

### 3.2 Performance evaluation of radiomics signatures

**Figure 3:**
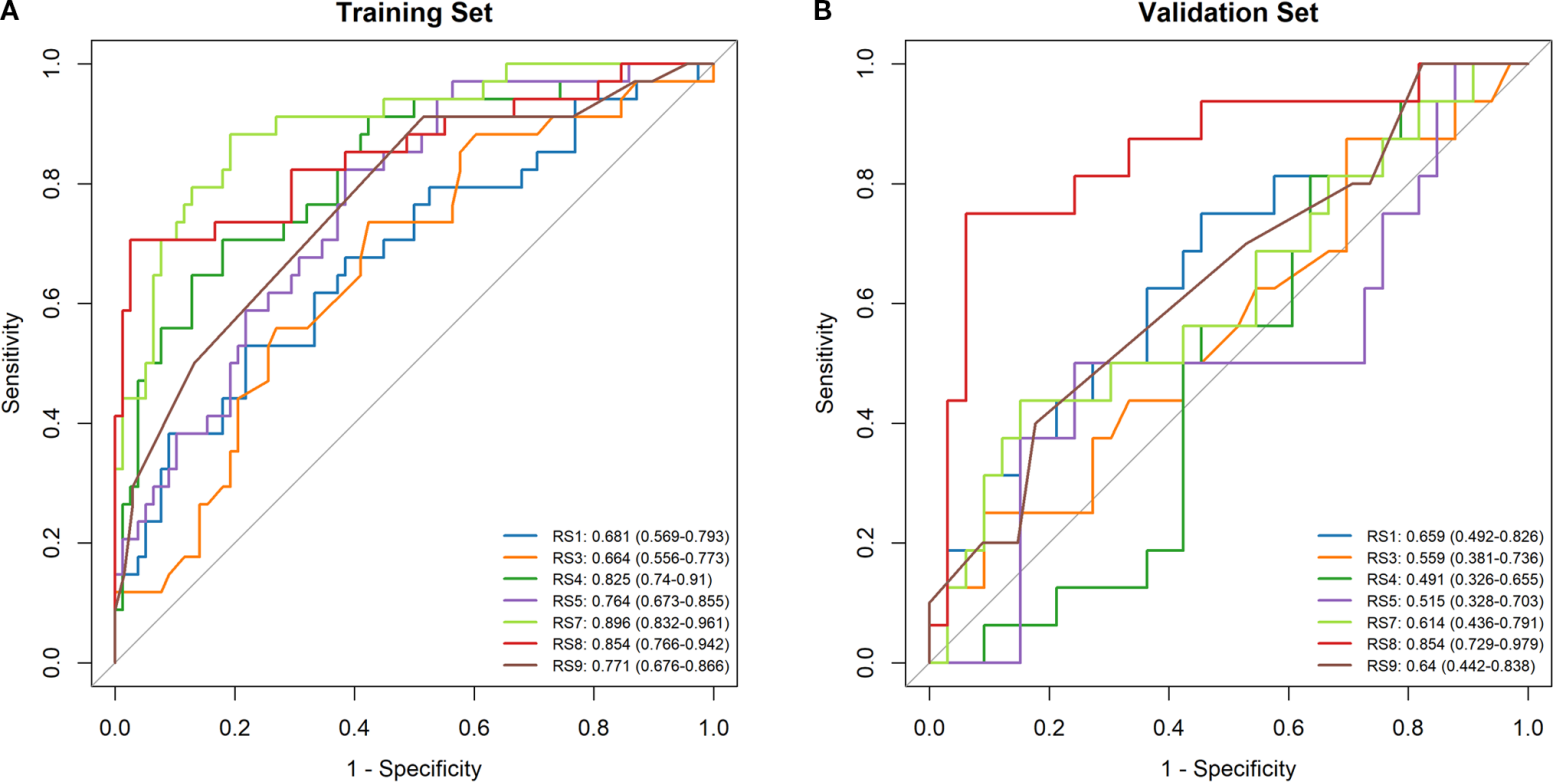
Comparison of ROC curve analyses in model based on logistic regression model. ROC curves of the RS1, RS3, RS4, RS5, RS7, RS8 and RS9 models in the training set **(A)**. ROC curves of the RS1, RS3, RS4, RS5, RS7, RS8 and RS9 models in the validation set **(B)**.

Radiomics features were extracted from planning CT scans across nine 5-Gy dose gradients (0–60 Gy), including the initial positioning CT (before radiotherapy) and a resetting CT (after a cumulative dose of 40–50 Gy), all within regions of interest (ROIs). No overlap with the PTV was found in the volumes receiving under 50Gy, while overlap was partial in the 50-55Gy and 55-60Gy dose ranges. LASSO regression identified significant radiomics signatures within the dose gradients of 5 Gy, 10–15 Gy, 15–20 Gy, 20–30 Gy, 40–50 Gy, 50–55 Gy, and 55–60 Gy, designated as RS1, RS3, RS4, RS5, RS7, RS8, and RS9, respectively. The differences in ΔRF features between the RP and non-RP groups were statistically significant across both the training and validation sets for these signatures. The formula for calculating each radiomics signature is available in the **Supplementary Materials Appendix S3**. The AUC values in the RS1, RS3, RS4, RS5, RS7, RS8 and RS9 in training set were 0.681, 0.664, 0.825, 0.764, 0.896 0.854 and 0.771, respectively (**Figure 3A**). The AUC values in the RS1, RS3, RS4, RS5, RS7, RS8 and RS9 in validation set were 0.659, 0.559,0.491,0.515,0.614,0.854 and 0.640, respectively (**Figure 3B**). Therefore, the RS8 model was identified as the most effective radiomics signature for this study. The results of the Delong’s test were presented in **Supplementary Table 2**.

### 3.3 Construction and comparison of combined model

**Figure 4:**
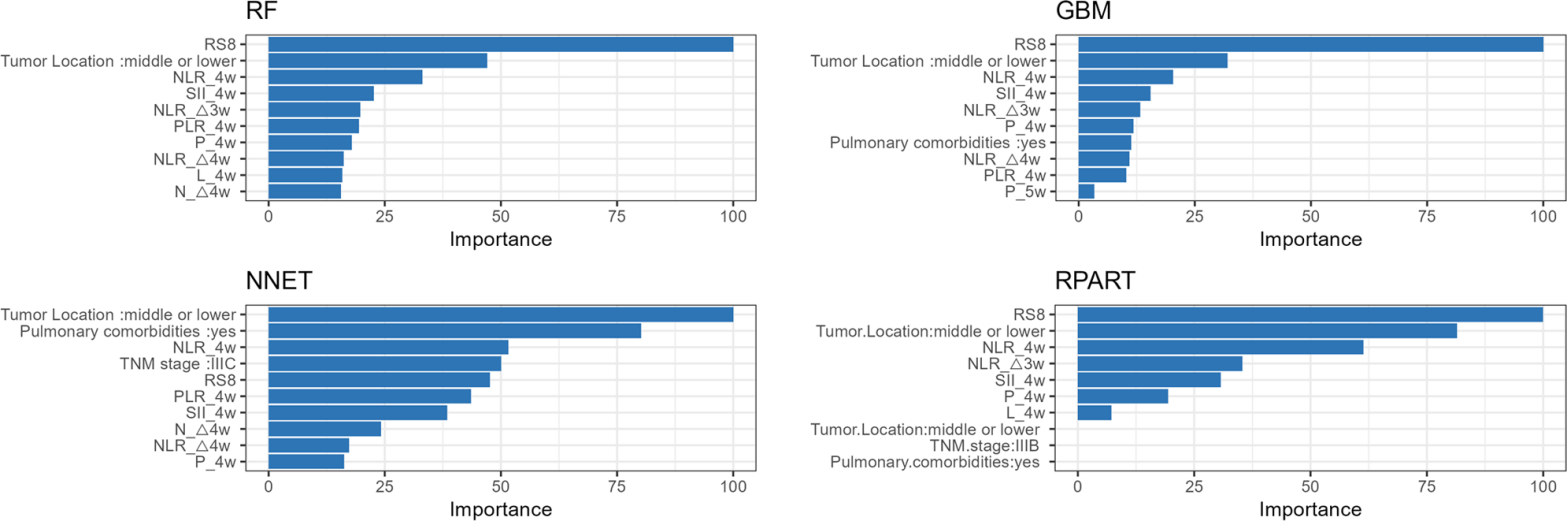
The variable influence factor ranking plots of RF, NNET, GBM and RPART models. RF, Random Forest; NNET, Neural Network; GBM, Gradient Boosting Machine; RPART, Recursive Partitioning and Regression Trees.

**Figure 5:**
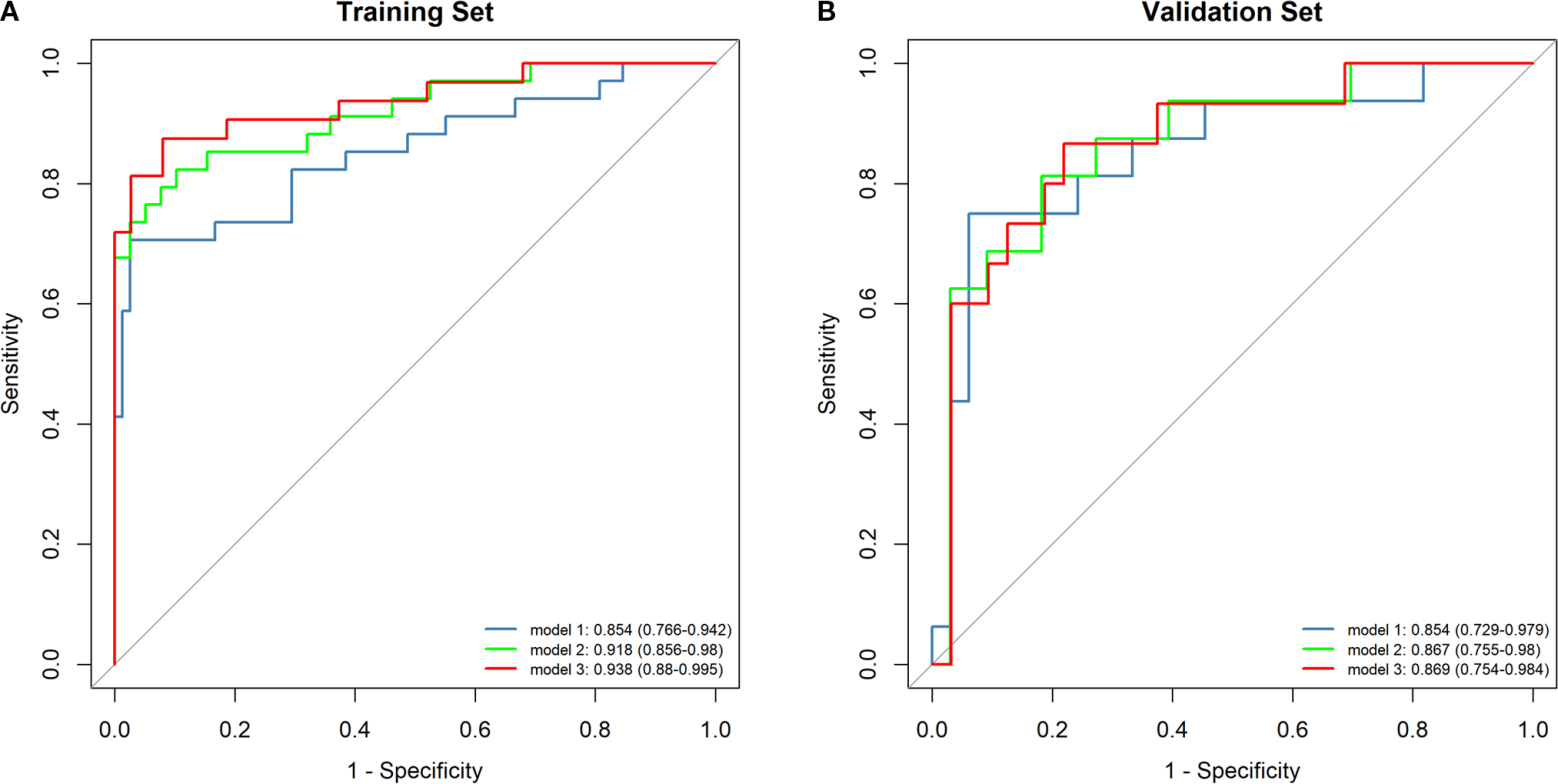
The ROC curves for the different models are depicted for both the training set **(A)** and the validation set **(B)**. Model1:RS8; Model2: RS8+ Tumor location; Model3: RS8+ Tumor location + NLR4w.

**Figure 6:**
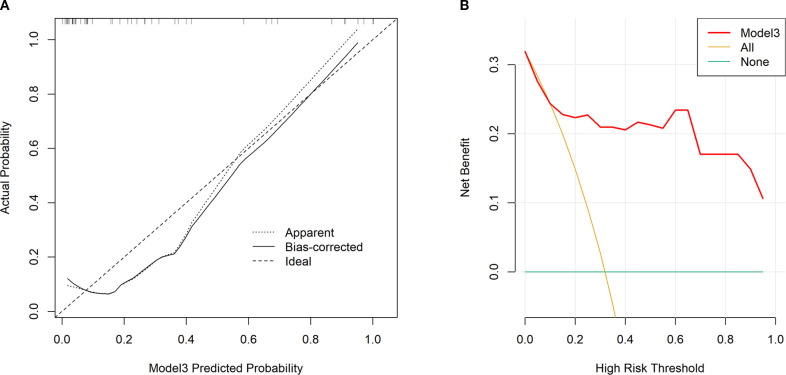
Calibration curves for our constructed model3 in validation set **(A)**. An ideal assessment was represented by the diagonal dotted line, whereas our nomogram performance was indicated by the remaining two lines. Decision curves for our constructed model3 that analyzed risk of RP≥2 in the validation set **(B)**. The y-axis stands for net benefit, whereas the red curve, horizontal blue line, and oblique orange line stand for nomogram, valid and invalid assumption, separately.

Tumor location, pulmonary comorbidities, TNM stage, RS8, N△4w, L4w, P4w, P5w, PLR4w, NLR4w, NLR△3w, NLR△4w, and SII4w were identified as 13 variables with p-values below 0.1 in the baseline characteristics (**Tables 1**, **2**). RS8, tumor location, and NLR4w were identified as the top three stable variables across the four machine learning algorithms (**Figure 4**). Logistic regression models for RS8, RS8 + tumor location, and RS8 + tumor location + NLR4w were designated as model 1, model 2, and model 3, respectively. ROC curves were generated to assess models’ predictive capability. Model 3 exhibited AUC values of 0.938 in the training set (**Figure 5A**) and 0.869 in the validation set (**Figure 5B**). In the training set, model 3 demonstrated superior performance compared to models 1 and 2, with higher AUC, sensitivity, accuracy, and negative predictive value (NPV) (**Table 3**). In the validation set, model 3 exhibited higher AUC, sensitivity, and NPV than models 1 and 2; however, its accuracy, specificity, and positive predictive value (PPV) were lower than those of models 1 and 2. Furthermore, the calibration curve revealed excellent agreement between the model3’s predictions and the actual RP observations within the validation cohort (**Figure 6A**). The DCA, depicted in **Figure 6B**, indicated that the model3 delivers substantial positive net benefits across a range of threshold probabilities up to 0.85 in the validation set. This showcases the model3’s valuable clinical utility in predicting RP risk.

**Table 3:** Predictive value of RP.

Performance Metric	Training set			Validation set		
	model1	model2	model3	model1	model2	model3
AUC	0.854	0.918	0.938	0.854	0.867	0.869
ACC	0.893	0.875	0.907	0.878	0.816	0.809
SE	0.706	0.824	0.875	0.750	0.812	0.867
SP	0.974	0.897	0.920	0.939	0.818	0.781
PPV	0.923	0.778	0.824	0.857	0.684	0.650
NPV	0.884	0.921	0.945	0.886	0.900	0.926

## 4 Discussion

This investigation delved into CT-based, dose distribution-oriented radiomics among LA-NSCLC patients receiving CCRT, employing machine learning classifier. It emerged that the LR-based radiomics feature model demonstrated robust performance. Remarkably, employing combined model markedly enhanced predictive power for RP.

In today**’**s advancing cancer immunotherapy landscape, precision radiotherapy represents a significant breakthrough in lung cancer treatment, particularly when combined with immunotherapy and other therapeutic strategies to boost survival rates for LA-NSCLC patients. However, the integration of ICI has raised concerns due to the increased incidence of pneumonia, noted in the PACIFIC study (2). Our study found an RP incidence rate of 27.45%, closely mirroring the 33.9% incidence reported in the PACIFIC trial. This highlights RP as a critical adverse reaction constraining the RT dose within the current LA-NSCLC treatment paradigm (19), underscoring the urgent need to discover new biomarkers beyond traditional clinical and dosimetric parameters.

The literature suggests the tumor**’**s location may affect radiotherapy-induced pulmonary toxicity. Meta-analyses and studies indicate that tumors situated in the middle or lower lung regions pose a higher RP risk than those located in the upper regions (5, 20, 21).This variation may be attributable to differences in radiosensitivity across lung areas and the physiological significance of perfusion and ventilation in the lower lung regions (8). Our study reaffirms tumor location as a risk factor for RP, emphasizing the critical nature of early preventative measures in RT planning for individuals at elevated risk.

In our analyses, pulmonary comorbidity was one of the top 10 significant variables in the variable importance rankings of the two machine learning algorithms, GBM and NNET, for predicting RP. The risk of RP can be influenced by pre-existing pulmonary comorbidities, including interstitial lung disease (ILD) and chronic obstructive pulmonary disease (COPD). Grade ≥r RP occurrence has been reported to significantly increase to 26% in patients with ILD on CT images, compared with 3% in patients with normal lungs (22). Additionally, Kimura**’**s study highlighted a notable increase in RP incidence correlating with the severity of emphysema, as defined by CT classification (23). This suggests that dose limitations derived from unselected patient populations may not be suitable for individuals with pre-existing pulmonary comorbidities.

Dosimetric parameters, including V20, V5, V30, and MLD, are well-established factors closely correlated with the incidence of RP. Barriger et al. noted a significant correlation between grade ≥2 RP and both MLD and V20 in populations treated with Stereotactic Body Radiotherapy (SBRT) (9). Furthermore, in patients with unresectable LA-NSCLC receiving consolidation durvalumab, various dosimetry parameters within the lungs and heart have been linked to pneumonitis (24). Despite analyzing dosimetric parameters, including V20, V5, V30, MLD, and the ratio of Planning Target Volume to Lung Volume (PTV/LV) in our study, no significant associations with RP were detected (all *p*> 0.05). However, this finding does not negate the potential importance of dosimetric factors in predicting RP risk.

Additionally, our findings suggest an increased susceptibility to RP in patients with elevated NLR levels at week 4. This association between NLR and pneumonia risk, including immune-related and pneumonia in patients with intracerebral hemorrhage, has been corroborated by other studies (25, 26). It underscores the necessity for future research to validate the most accurate inflammatory markers for RP prediction, even though they did not show a significant impact in our study.

To our knowledge, this is the first study to employ CT-based dose-segmentation features for predicting RP in the context of immune consolidation therapy following CCRT in LA-NSCLC patients. Our approach finds indirect support from previous research. For instance, Liang et al. advocated for using ipsilateral, contralateral, and whole lung ROIs for RP prediction, noting that their dosiomics model (AUC=0.782) outperformed both the dosimetric (AUC=0.676) and NTCP models (AUC=0.744) (15). Similarly, Adachi et al. focused on dose-segmented dosimetric characteristics within the Vx Gy area, highlighting that unirradiated lung regions might be irrelevant for RP prediction. Their findings supported texture-based dosiomic features as effective RP predictors (16). Zhang et al. also observed that combining radiomics, dosiomics, and clinical variables could enhance the accuracy of RP occurrence predictions (27).

In our study, we extracted features from dose-segmented regions, refining the dose segmentation range compared to Adachi et al.’s approach and analyzing features from dose ranges of 0–5 Gy up to 55–60 Gy. We established radiomics and dosiomics signature models by comparing features extracted across different dose regions, identifying the 50–55 Gy (RS8) model as providing the best predictive performance. This superior prediction may be linked to the notable increase in feature change values with escalating RT dose (28). Our results suggest the importance of meticulous target area delineation in patients with elevated RP risk factors in clinical practice, aiming to minimize damage to healthy lung tissue.

Diverging from the study by Adachi et al., our approach sought to augment RP prediction accuracy by monitoring dynamic changes in radiomic features from the initial positioning CT to the subsequent resetting CT during radiotherapy. This concept aligns with findings from Cunliffe et al., who demonstrated the predictive superiority of evaluating changes in texture features before and after RT in CT images over reliance on single time-point metrics (28). The introduction of a novel delta radiomics signature, delta-RF = (RF_CT2_ - RF_CT1_)/RF_CT1_ × 100%, by Wang et al. further emphasizes the value of dynamic feature analysis. This method has proven effective in enhancing RP detection capabilities, underscoring the importance of dynamic variations in radiomic features at multiple time points for improving RP prediction accuracy beyond the limitations of static image data (17).

The limitations of our study are noteworthy. Primarily, the retrospective nature of this research and the absence of pulmonary function data for a third of the participants hindered a more detailed clinical assessment of COPD classification and a precise RP risk scoring. Secondly, being a single-institution study with a relatively small sample size and lacking an external validation set, our findings are potentially susceptible to selection bias. Despite not identifying a significant correlation between RP and dosimetric parameters, it emphasizes the necessity for broader multi-institutional studies to re-evaluate the predictive relevance of dosimetry and dosiomics for RP in the context of LA-NSCLC during the era of immunotherapy. Furthermore, the variability of ΔRF could be influenced by the use of different imaging histology software packages and processing methodologies. Advancing towards the standardization of the entire radiomics workflow is an essential step for improving the precision of RP predictions.

## 5 Conclusions

Our study demonstrates that employing dose distribution-based radiomics significantly enhances the ability to predict grade ≥2 RP. Furthermore, we have introduced a novel combined model that integrates radiomics features with clinicopathological parameters and hematological inflammatory markers. The advent of this comprehensive model offers a valuable tool for clinicians, enhancing their ability to monitor RP risk and tailor treatment strategies accordingly. This proactive approach is pivotal in mitigating RP risk, ultimately contributing to the optimization of patient care in the treatment of lung cancer.

## Data Availability

The raw data supporting the conclusions of this article will be made available by the authors, without undue reservation.
