# Supplementary material for: Predicting radiation pneumonitis with dose-segmented radiomics in locally advanced non- small cell lung cancer patients undergoing consolidative immunotherapy post- concurrent chemoradiotherapy
Source: Front Immunol. 2025 Sep 26;16:1684629. doi: 10.3389/fimmu.2025.1684629 (PMC12510859; doi:10.3389/fimmu.2025.1684629)
Supplement: Supplementary file 1 [file Table1.docx]

**TABLE S1** **The types of radiomics features and subclass features studied in our work.**

| **Shape** | **First-order** | **GLCM** | **GLRLM** | **GLSZM** | **GLDM** | **NGTDM** |
| --- | --- | --- | --- | --- | --- | --- |
| Elongation | 10Percentile | Autocorrelation | GrayLevelNonUniformity | GrayLevelNonUniformity | DependenceEntropy | Busyness |
| Flatness | 90Percentile | ClusterProminence | GrayLevelNonUniformityNormalized | GrayLevelNonUniformityNormalized | DependenceNonUniformity | Coarseness |
| LeastAxisLength | Energy | ClusterShade | GrayLevelVariance | GrayLevelVariance | DependenceNonUniformityNormalized | Complexity |
| MajorAxisLength | Entropy | ClusterTendency | HighGrayLevelRunEmphasis | HighGrayLevelZoneEmphasis | DependenceVariance | Contrast |
| Maximum2DDiameterColumn | InterquartileRange | Contrast | LongRunEmphasis | LargeAreaEmphasis | GrayLevelNonUniformity | Strength |
| Maximum2DDiameterRow | Kurtosis | Correlation | LongRunHighGrayLevelEmphasis | LargeAreaHighGrayLevelEmphasis | GrayLevelVariance |  |
| Maximum2DDiameterSlice | Maximum | DifferenceAverage | LongRunLowGrayLevelEmphasis | LargeAreaLowGrayLevelEmphasis | HighGrayLevelEmphasis |  |
| Maximum3DDiameter | MeanAbsoluteDeviation | DifferenceEntropy | LowGrayLevelRunEmphasis | LowGrayLevelZoneEmphasis | LargeDependenceEmphasis |  |
| MeshVolume | Mean | DifferenceVariance | RunEntropy | SizeZoneNonUniformity | LargeDependenceHighGrayLevelEmphasis |  |
| MinorAxisLength | Median | Id | RunLengthNonUniformity | SizeZoneNonUniformityNormalized | LargeDependenceLowGrayLevelEmphasis |  |
| Sphericity | Minimum | Idm | RunLengthNonUniformityNormalized | SmallAreaEmphasis | LowGrayLevelEmphasis |  |
| SurfaceArea | Range | Idmn | RunPercentage | SmallAreaHighGrayLevelEmphasis | SmallDependenceEmphasis |  |
| SurfaceVolumeRatio | RobustMeanAbsoluteDeviation | Idn | RunVariance | SmallAreaLowGrayLevelEmphasis | SmallDependenceHighGrayLevelEmphasis |  |
| VoxelVolume | RootMeanSquared | Imc1 | ShortRunEmphasis | ZoneEntropy | SmallDependenceLowGrayLevelEmphasis |  |
|  | Skewness | Imc2 | ShortRunHighGrayLevelEmphasis | ZonePercentage |  |  |
|  | TotalEnergy | InverseVariance | ShortRunLowGrayLevelEmphasis | ZoneVariance |  |  |
|  | Uniformity | JointAverage |  |  |  |  |
|  | Variance | JointEnergy |  |  |  |  |
|  |  | JointEntropy |  |  |  |  |
|  |  | MCC |  |  |  |  |
|  |  | MaximumProbability |  |  |  |  |
|  |  | SumAverage |  |  |  |  |
|  |  | SumEntropy |  |  |  |  |
|  |  | SumSquares |  |  |  |  |

GLCM gray level cooccurence matrix; GLDM gray level dependence matrix; GLRLM gray level run length matrix; GLSZM gray level size zone matrix

**FIGURE S1**

**
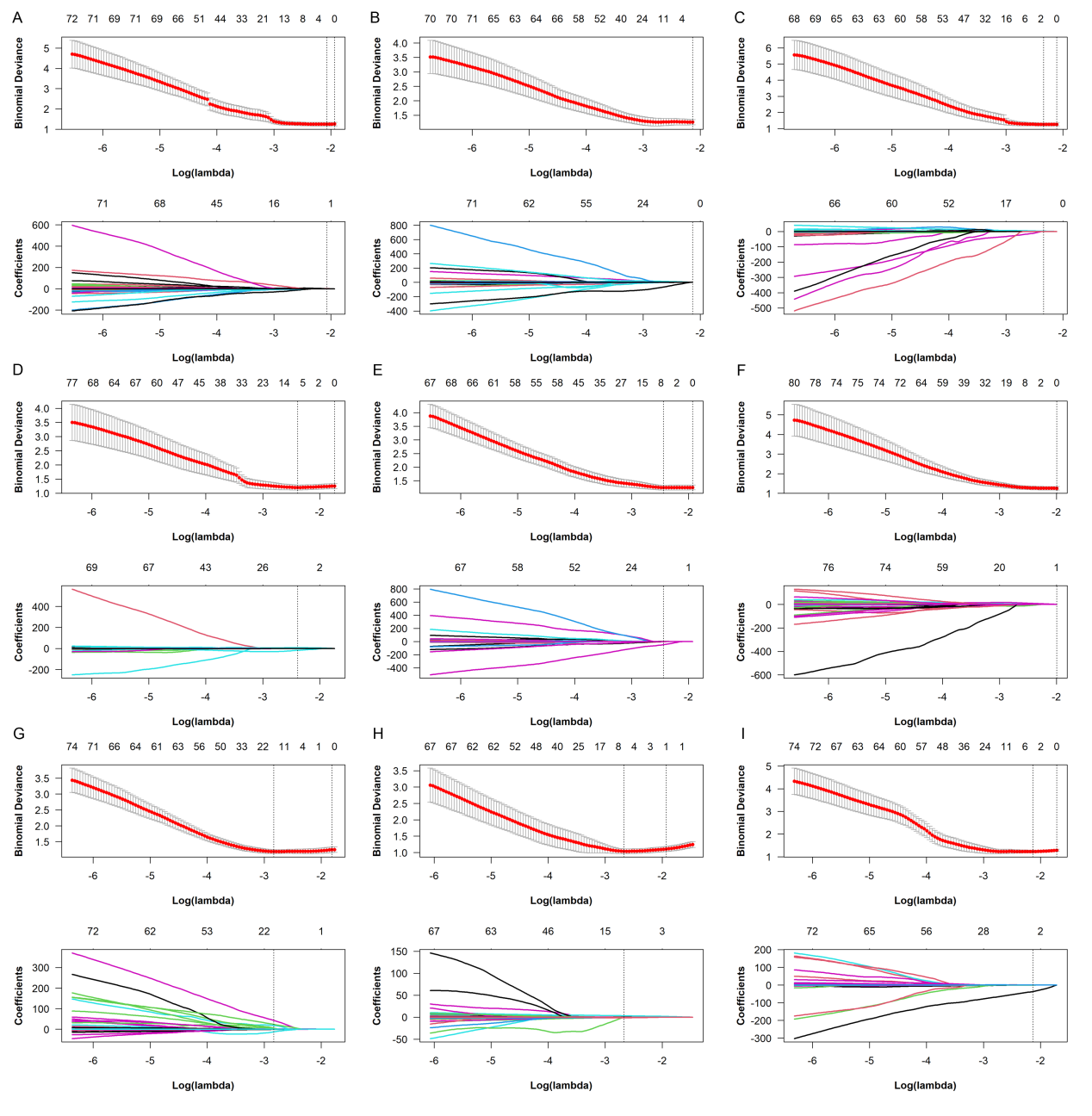
**

**Feature selection using the least absolute shrinkage and selection operator (LASSO) algorithm.**

Turning optimal parameter lambda (λ) using 10-fold cross-validation and minimum criterion in Lasso model in

the training set. The left and right dashed lines represent the minimum criterion and the 1-standard error (1-SE) criterion, respectively. The 1-SE criterion has been applied. LASSO coefficients profile plot with different log (λ) was shown in the training set. Lasso model in the training set of ROI(1) of dose 0-5 gy is picture a and the optimal λ value is 0.1257642. Lasso model in the training set of ROI(2) of dose 5-10 gy is picture b and the optimal λ value is 0.1186546. Lasso model in the training set of ROI(3) of dose 10-15 gy is picture c and the optimal λ value is 0.09634795. Lasso model in the training set of ROI(4) of dose 15-20 gy is picture d and the optimal λ value is 0.09146011. Lasso model in the training set of ROI(5) of dose 20-30 gy is picture e and the optimal λ value is 0.08716486. Lasso model in the training set of ROI(6) of dose 30-40 gy is picture f and the optimal λ value is 0.1358363. Lasso model in the training set of ROI(7) of dose 40-50 gy is picture g and the optimal λ value is 0.05873212. Lasso model in the training set of ROI(8) of dose 50-55 gy is picture h and the optimal λ value is 0.06903612. Lasso model in the training set of ROI(9) of dose 55-60 gy is picture i and the optimal λ value is 0.1183852.

**Appendix S3**

RS1 = -0.8617 + 5.1306 ×wavelet-LLH-firstorder-10Percentile

RS3 = -0.80 + 11.41 ×wavelet-HHH-glszm-SmallAreaEmphasis

RS4 = -0.52635 +12.30338 ×wavelet-LLH-glrlm-RunEntropy

-0.08641 ×wavelet-HLL-glcm-ClusterShade

+13.11650 ×wavelet-LLL-glcm-SumEntropy

-73.47155 × squareroot-glcm-Idmn

-3.66522 × exponential-glszm-ZoneEntropy

RS5 = -0.8891 -51.5452 × gradient-glcm-Idn

+1.1144 × wavelet-LLH-firstorder-RobustMeanAbsoluteDeviation

-26.0224 × wavelet-LHL-glcm-Idn

-1874.8810 × wavelet-LLL-glcm-Idmn

-5.1622 × square-glrlm-GrayLevelNonUniformityNormalized

-0.6118 × square-glszm-LargeAreaHighGrayLevelEmphasis

RS7 = -0.66470 +50.96917 × gradient-gldm-DependenceEntropy

+2.87372 × lbp-2D-glszm-SizeZoneNonUniformity

+5.85323 × wavelet-LLH-firstorder-10Percentile

+0.31654 × wavelet-LLH-firstorder-RobustMeanAbsoluteDeviation

+394.90403 × wavelet-LLH-glcm-Idmn

-48.96635 × wavelet-LHL-glcm-Idmn

+109.63073 × wavelet-LHH-glcm-Idmn

-0.20168 × wavelet-HLL-firstorder-Skewness

+190.78274× wavelet-HHH-glcm-Idmn

+0.03862× wavelet-LLL-glszm-LargeAreaHighGrayLevelEmphasis

+1.28366× wavelet-LLL-ngtdm-Contrast

-0.45640× square-glszm-LargeAreaHighGrayLevelEmphasis

-148.45388× squareroot-glcm-Idmn

-0.71362× squareroot-glcm-MaximumProbability

-9.68506× logarithm-firstorder-Median

+227.84960× exponential-glrlm-GrayLevelNonUniformityNormalized

RS8 = -1.6354 +1.2547 × original-ngtdm-Contrast

+7.4998 × wavelet-LLH-firstorder-10Percentile

-0.2522 × wavelet-LLH-glcm-ClusterShade

+1.4811 × wavelet-HHL-firstorder-Kurtosis

-2.7029 × square-glrlm-GrayLevelNonUniformityNormalized

+0.6775 × square-ngtdm-Busyness

RS9 = -0.6669 - 3.8174 × wavelet-LLH-glszm-GrayLevelNonUniformityNormalized

-146.9358 × logarithm-glcm-Idmn

**TABLE S2 Results of Delong's test for the RS model.**

| **Group** | **Comparison** | **AUC Difference** | **P value** | **FDR Adjusted P-value** |
| --- | --- | --- | --- | --- |
| Training Set | RS1 vs RS7 | 0.215 | <0.001 | <0.001 |
| Training Set | RS1 vs RS8 | 0.172 | <0.001 | 0.003 |
| Training Set | RS3 vs RS7 | 0.232 | <0.001 | 0.004 |
| Training Set | RS5 vs RS7 | 0.132 | 0.008 | 0.039 |
| Training Set | RS3 vs RS8 | 0.189 | 0.009 | 0.039 |
| Training Set | RS1 vs RS4 | 0.144 | 0.013 | 0.046 |
| Training Set | RS3 vs RS4 | 0.161 | 0.023 | 0.070 |
| Training Set | RS7 vs RS9 | 0.125 | 0.033 | 0.087 |
| Training Set | RS5 vs RS8 | 0.090 | 0.124 | 0.255 |
| Training Set | RS3 vs RS5 | 0.100 | 0.129 | 0.255 |
| Training Set | RS1 vs RS5 | 0.083 | 0.134 | 0.255 |
| Training Set | RS3 vs RS9 | 0.107 | 0.147 | 0.258 |
| Training Set | RS4 vs RS7 | 0.071 | 0.163 | 0.264 |
| Training Set | RS8 vs RS9 | 0.083 | 0.211 | 0.316 |
| Training Set | RS1 vs RS9 | 0.090 | 0.232 | 0.325 |
| Training Set | RS4 vs RS5 | 0.061 | 0.247 | 0.325 |
| Training Set | RS7 vs RS8 | 0.043 | 0.377 | 0.466 |
| Training Set | RS4 vs RS9 | 0.054 | 0.406 | 0.474 |
| Training Set | RS4 vs RS8 | 0.029 | 0.566 | 0.626 |
| Training Set | RS1 vs RS3 | 0.017 | 0.809 | 0.850 |
| Training Set | RS5 vs RS9 | 0.007 | 0.917 | 0.917 |
| Validation Set | RS5 vs RS8 | 0.339 | 0.002 | 0.022 |
| Validation Set | RS4 vs RS8 | 0.364 | 0.002 | 0.022 |
| Validation Set | RS3 vs RS8 | 0.295 | 0.003 | 0.023 |
| Validation Set | RS7 vs RS8 | 0.241 | 0.004 | 0.023 |
| Validation Set | RS1 vs RS8 | 0.195 | 0.011 | 0.046 |
| Validation Set | RS8 vs RS9 | 0.214 | 0.077 | 0.269 |
| Validation Set | RS1 vs RS5 | 0.144 | 0.153 | 0.458 |
| Validation Set | RS1 vs RS4 | 0.169 | 0.218 | 0.571 |
| Validation Set | RS4 vs RS9 | 0.149 | 0.259 | 0.605 |
| Validation Set | RS5 vs RS7 | 0.098 | 0.294 | 0.617 |
| Validation Set | RS4 vs RS7 | 0.123 | 0.350 | 0.653 |
| Validation Set | RS5 vs RS9 | 0.125 | 0.373 | 0.653 |
| Validation Set | RS1 vs RS3 | 0.100 | 0.431 | 0.696 |
| Validation Set | RS3 vs RS4 | 0.068 | 0.510 | 0.766 |
| Validation Set | RS3 vs RS9 | 0.081 | 0.552 | 0.773 |
| Validation Set | RS1 vs RS7 | 0.045 | 0.591 | 0.776 |
| Validation Set | RS3 vs RS7 | 0.055 | 0.715 | 0.883 |
| Validation Set | RS3 vs RS5 | 0.044 | 0.780 | 0.884 |
| Validation Set | RS7 vs RS9 | 0.026 | 0.848 | 0.884 |
| Validation Set | RS4 vs RS5 | 0.025 | 0.868 | 0.884 |
| Validation Set | RS1 vs RS9 | 0.019 | 0.884 | 0.884 |
